# Using GHQ-12 to Screen Mental Health Issues in People with Emphysema

**DOI:** 10.3390/healthcare11142009

**Published:** 2023-07-12

**Authors:** Weixi Kang, Edward Whelan, Sònia Pineda Hernández

**Affiliations:** 1 UK DRI Care Research and Technology Centre, Department of Brain Sciences, Imperial College London, London W12 0BZ, UK; 2 Independent Researcher, 99MX QH Maynooth, Ireland; 3 School of Health Sciences, University Pompeu Fabra, 08002 Barcelona, Spain

**Keywords:** GHQ-12, mental health, emphysema, anxiety, depression, anhedonia

## Abstract

Although previous studies have investigated the presence of psychometric comorbidities in individuals with emphysema, our understanding of the impact of emphysema on general mental health and specific dimensions of mental health, such as social dysfunction and anhedonia, depression and anxiety, and loss of confidence, remains limited. This research aims to examine the influence of emphysema on general mental health and its dimensions using the GHQ-12 assessment. By employing confirmatory factor analysis (CFA), the predictive normative approach, and one-sample *t*-test, we analyzed data from Understanding Society: the UK Household Longitudinal Study (UKHLS), including 97 individuals clinically diagnosed with emphysema and 8980 individuals without a clinical diagnosis of emphysema. The findings of this study indicate that individuals with clinically diagnosed emphysema experience poorer general mental health (t(96) = 8.41, *p* < 0.001, Cohen’s d = 0.12, 95% C.I. [0.09, 0.15]), increased levels of social dysfunction and anhedonia (t(96) = 6.02, *p* < 0.001, Cohen’s d = 0.09, 95% C.I. [0.06, 0.11]), heightened depression and anxiety (t(96) = 7.26, *p* < 0.001, Cohen’s d = 0.11, 95% C.I. [0.08, 0.14]), as well as elevated loss of confidence (t(96) = 6.40, *p* < 0.001, Cohen’s d = 0.09, 95% C.I. [0.07, 0.12]). These findings suggest the need for intervention programs aimed at improving the mental health of individuals with emphysema.

## 1. Introduction

Emphysema is a chronic and progressive lung disease characterized by the irreversible destruction of the alveoli, leading to decreased lung function, impaired oxygen exchange, and difficulty in breathing. As part of a group of chronic, obstructive, and typically progressive lung conditions known as COPD, emphysema can impact mental health. According to the World Health Organization (WHO) [1], mental health refers to a state of well-being in which individuals can achieve their potential, effectively cope with everyday stressors, engage in productive work, and contribute to their community. COPD patients with emphysema are more likely to have other comorbidities compared to the general population, including lung cancer, psychological disorders, high blood pressure, diabetes mellitus, kidney failure, osteoporosis, skeletal muscle dysfunction, and cardiovascular diseases (such as ischemic heart disease, heart failure, or stroke), often accompanied by mental health conditions such as depression and anxiety. These concurrent chronic conditions pose challenges for diagnosis and treatment, further complicating the prognosis and necessitating active identification [2]. Moreover, prompt treatment is crucial as these comorbidities independently increase mortality and hospitalization rates in patients with COPD [3]. Psychological illnesses, including anxiety and depression, are more prevalent among patients with COPD compared to the general population [4]. However, distinguishing between anxiety and the dyspnea characteristic of COPD can be challenging, potentially leading to underdiagnosis [5]. Given the high risk of mortality associated with anxiety, particularly in women with existing COPD diagnoses, early treatment is essential [6]. Depression is also more common among patients with COPD than the general population, especially among those utilizing home oxygen therapy programs, with rates reaching up to 60% [7]. Such individuals often exhibit poor adherence to medical treatment, reduced physical activity, and diminished quality of life. Untreated anxiety and depression in patients with COPD have been linked to various adverse effects [8]. Previous studies have demonstrated that patients with COPD and with comorbid anxiety and depression are more susceptible to exacerbations and hospitalization compared to those with COPD alone. They also experience more symptoms, greater impairment, and lower social functioning [8,9,10]. Furthermore, depression has been identified as a potential risk factor for mortality in patients with COPD [10]. Other investigations have also highlighted the significance of the coexistence of COPD and psychological distress. Psychological changes can hinder patients’ ability to manage physical symptoms, leading to a sense of weakness and exacerbating their psychological state [11].

The General Health Questionnaire (GHQ), developed in the 1970s, has gained recognition as a reliable and valid tool for assessing mental well-being. Among the various versions of this questionnaire, the GHQ-12 is the most commonly utilized, consisting of 12 items to evaluate mental health [11]. Extensive research examining the psychometric properties of the GHQ-12 [10,12,13,14,15,16] has consistently shown its accuracy, specificity, and reliability [17,18]. Although initially designed as a unidimensional scale, researchers have investigated the factor structure of the GHQ-12. Notably, a substantial body of evidence supports the adoption of a 3-factor model [19,20,21,22,23,24,25], comprising GHQ-12A (social dysfunction and anhedonia; six items), GHQ-12B (anxiety and depression; four items), and GHQ-12C (loss of confidence; two items). The strong interrelation among these factors serves as an argument in favor of using the GHQ-12’s unidimensional model over the factor solution [19,20,23]. However, it is important to note that employing a simplistic framework may potentially inflate correlations between modeled factors and simulated data [26]. Griffith and Jones (2019) caution against embracing unidimensionality solely based on these associations, as it may lead to a self-fulfilling prophecy of oversimplification [27]. Given the ongoing debate regarding the merits of the two approaches, the present study considers both the GHQ-12’s unidimensional structure and its 3-factor multidimensional structure.

Hence, although certain studies have explored the influence of emphysema on psychiatric disorders, there is a dearth of knowledge regarding the specific impact of clinically diagnosed emphysema on the various aspects of mental health measured by the factors encompassed in the GHQ-12 questionnaire, including social dysfunction and anhedonia, anxiety and depression, and loss of confidence.

Consequently, the objective of this study is to examine the effects of emphysema on social dysfunction and anhedonia, anxiety and depression, and loss of confidence.

## 2. Methods

### 2.1. Data

Data for this study were obtained from Understanding Society, an ongoing project conducted at the University of Essex [28], which has been collecting data from participating households since 1991. Specifically, we analyzed data from Wave 10, collected between 2018 and 2019 [28]. The University of Essex Ethics Committee granted approval for all data collection procedures. Prior to participating in the study, all participants provided informed consent by signing a consent form. Individuals with any missing variables were excluded from the analysis, resulting in a final sample of 97 individuals with clinically diagnosed emphysema and 8980 individuals without clinically diagnosed emphysema.

### 2.2. Measures

#### 2.2.1. Emphysema

Participants were asked the following question to indicate that if they have been clinically diagnosed with emphysema, “Has a doctor or other health professional ever informed you that you have any of these conditions? Emphysema”. Self-reported emphysema can serve as a valid tool for measuring emphysema in cohort-based studies, as demonstrated in previous research [29].

#### 2.2.2. Mental Mealth

Mental health scores were calculated using the GHQ-12, a 12-item unidimensional measure of mental health [11]. The GHQ-12 utilized a Likert scale ranging from 0 (“Not at all”) to 3 (“Much more than usual”) for rating purposes. To assess the overall mental health status, a summative score across all 12 items was computed, with higher scores indicating poorer mental health. For the factor analysis, the GHQ-12 was graded on a scale of 1 (“Not at all”) to 4 (“Much more than usual”).

#### 2.2.3. Demographic Controls

In the model, several demographic variables were considered as controls, including age, sex, monthly income, highest level of education attained, legal marital status, and place of residence. The coding scheme employed for these variables is outlined in Table 1.

### 2.3. Statistical Models

The GHQ-12, a measure of mental health, was subjected to a confirmatory factor analysis (CFA) to establish a 3-factor structure. Subsequently, we utilized a predictive normative modeling approach to investigate the impact of clinically diagnosed emphysema on general mental health and its dimensions. To predict mental health outcomes for individuals without a clinical diagnosis of emphysema, we developed four generalized models that incorporated demographic variables (i.e., age, sex, monthly income, highest level of education attained, legal marital status, and place of residence) of health controls as predictors. Subsequently, using these trained models, we projected the expected general and specific aspects of mental health for individuals with clinically diagnosed emphysema. To assess the disparities between actual mental health scores and expected scores in patients with confirmed emphysema, we conducted one-sample *t*-tests. This method was preferred over a paired-sample *t*-test due to its ability to account for demographic factors. MATLAB 2018a (MathWorks, Natick, MA, USA) was employed for all statistical analyses.

## 3. Results

**Figure 1:**
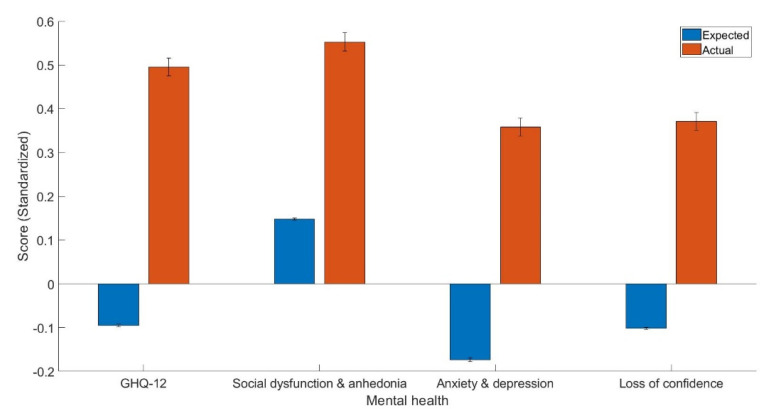
The expected and actual general (GHQ-12) and dimensions of mental health scores including GHQ-12A (social dysfunction and anhedonia), GHQ-12B (anxiety and depression), and GHQ-12C (loss of confidence).

Table 1 provides descriptive statistics for the variables under investigation. Confirmatory factor analysis (CFA) identified three variables: GHQ-12A, consisting of six items related to social dysfunction and anhedonia; GHQ-12B, comprising four items pertaining to anxiety and depression; and GHQ-12C, consisting of two items measuring loss of confidence. Table 2 presents the loadings for these items. Additionally, Table 3 displays the estimates of the variables utilized in the linear models developed using healthy controls. Importantly, individuals with clinically diagnosed emphysema exhibited significantly poorer general mental health (t(96) = 8.41, *p* < 0.001, Cohen’s d = 0.12, 95% C.I. [0.09, 0.15]; Figure 1), higher levels of social dysfunction and anhedonia (t(96) = 6.02, *p* < 0.001, Cohen’s d = 0.09, 95% C.I. [0.06, 0.11]), increased anxiety and depression (t(96) = 7.26, *p* < 0.001, Cohen’s d = 0.11, 95% C.I. [0.08, 0.14]), as well as greater loss of confidence (t(96) = 6.40, *p* < 0.001, Cohen’s d = 0.09, 95% C.I. [0.07, 0.12]).

## 4. Discussion

The objective of this study was to examine the impact of emphysema on general mental health and its specific dimensions, as assessed by the GHQ-12. Confirmatory factor analysis (CFA) was utilized to identify three components: GHQ-12A (consisting of six items measuring social dysfunction and anhedonia), GHQ-12B (comprising four items measuring anxiety and depression), and GHQ-12C (including two items measuring loss of confidence). The three-factor structure found in this study aligns with previous research that identified three elements within the GHQ-12 structure [10,12,13,14,15,16]. The factor loadings observed in the current investigation were notably high, as presented in Table 2.

Our observations indicate that individuals with emphysema encounter a broader range of mental health issues. These findings align with previous studies, which have also identified social dysfunction and anhedonia as common features among emphysema patients [9,10]. The physical health challenges associated with emphysema may contribute to disrupted social relationships [30]. Additionally, our results support prior research that has documented a higher prevalence of depression and anxiety symptoms in individuals with emphysema [9,10]. Notably, our study reveals a novel finding that individuals with emphysema exhibit greater loss of confidence compared to healthy controls. Although previous research has reported decreased confidence in general physicians following an emphysema diagnosis [31], the positive association between self-confidence and quality of life in women with breast emphysema further highlights the significance of this finding as people with emphysema tend to have poorer quality of life and thus lower self-confidence [32].

Several potential explanations can account for the observed associations between emphysema and mental health. A recent systematic review and meta-analysis of 25 long-term follow-up studies revealed a likely bidirectional relationship between COPD and depression [5], suggesting that depression may both contribute to and result from COPD. However, the specific pathways linking COPD to feelings of sadness and anxiety remain uncertain. It is unclear whether smoking, anxiety, or depression are directly linked to COPD. Smoking escalates the risk and severity of COPD, making daily tasks challenging and stressful, and increasing the likelihood of experiencing depression or anxiety in patients with COPD. Confounding factors such as previous tobacco use and nicotine dependence appear to account for a significant portion of the associations between anxiety disorders and COPD [7]. However, nicotine dependence seems to play a substantial role in the relationship between mood disorders and COPD. Therefore, these cross-sectional associations do not establish causality but underscore the need for well-designed research studies. Depression and anxiety can perpetuate feelings of anxiety and despair by inducing fear, panic, hopelessness, low self-esteem, social isolation, and dependency on caregivers. Emerging research suggests that low-grade chronic inflammation may serve as a potential mediator of the link between depressive symptoms and pulmonary function. Both COPD and late-life depression have been associated with elevated levels of inflammatory markers [8,9]. A recent study involving a community sample of older individuals found that increased levels of inflammatory biomarkers such as interleukin-6 and C-reactive protein partially accounted for the correlation between depressive symptoms and lung obstruction [10]. Finally, the associations between emphysema and mental health may be explained by biological, behavioral, and social factors. For instance, the prevalence of depression was examined in various groups, including patients with COPD (*n* = 2118), smokers without COPD (*n* = 335), and nonsmokers without COPD (*n* = 243) [11]. Depression rates were found to be 26% among patients with COPD, 12% among smokers, and 7% among nonsmokers. Thus, smoking may have the potential and mediate the association between emphysema and mental health.

Several limitations should be acknowledged in our study. Firstly, it is important to note that this research follows a cross-sectional design, which poses challenges in establishing causal relationships. To overcome this limitation and provide stronger evidence, future studies are advised to employ a longitudinal approach. Secondly, the data utilized in this study relied on self-report measures, which introduces the potential for bias. To enhance the validity and reliability of findings, it is recommended that future research incorporates objective assessments. Finally, it is worth mentioning that the UKHLS did not collect information regarding the specific type of emphysema among participants. This omission is significant as the type of emphysema may have varying effects on mental health. Hence, future investigations should take into account the distinct subtypes of emphysema to gain a more comprehensive understanding of their impact on mental well-being.

## 5. Conclusions

In summary, emphysema has a significant impact on various aspects of mental health. Individuals with emphysema are more susceptible to experiencing multiple mental health challenges, including social dysfunction and anhedonia, anxiety and depression, and loss of confidence. Mental health comorbidities in patients with emphysema can lead to severe consequences, depleting both their own and their caregivers’ coping mechanisms and potentially increasing their healthcare utilization. Interventions and preventions should be developed to reduce mental health issues in people with emphysema. Future studies should also evaluate the effectiveness of other inventories besides GHQ-12 in measuring mental health in people with emphysema.

## Figures and Tables

**Table 1:** The demographic characteristics of healthy controls and people with emphysema.

	Healthy Controls		People with Emphysema	
Variables	Mean	S.D.	Mean	S.D.
Age	68.01	8.52	68.31	10.56
Monthly income (£)	1708.70	1458.77	1248.03	651.68
	N	%	N	%
**Sex**				
Male	4612	51.36	50	51.55
Female	4368	48.64	47	48.45
**Highest educational qualification**				
Below college	6446	71.78	88	70.72
College	2534	28.22	9	9.28
**Legal marital status**				
Single	3235	36.02	58	59.79
Married	5745	63.98	39	40.21
**Residence**				
Urban	6354	70.76	74	76.29
Rural	2626	29.24	23	23.71

**Table 2:** The factor loadings for the GHQ-12’s three-factor structure.

GHQ-12 Items	GHQ-12A (Social Dysfunction and Anhedonia; 6 Items)	GHQ-12B (Anxiety and Depression; 4 Items)	GHQ-12C (Loss of Confidence; 2 Items)
Concentration	0.53	0.25	−0.10
Loss of sleep	0.00	0.66	0.05
Playing a useful role	0.67	−0.16	0.16
Constantly under strain	0.79	−0.14	0.00
Problem overcoming difficulties	−0.01	0.88	−0.07
Unhappy or depressed	0.08	0.56	0.21
Losing confidence	0.68	0.22	−0.15
Believe worthless	0.71	−0.03	0.04
General happiness	0.03	0.53	0.34
Capable of making decisions	0.00	0.23	0.70
Ability to face problems	0.10	0.02	0.74
Enjoy day-to-day activities	0.56	0.10	0.10

**Table 3:** The estimates (b) of linear models trained using demographic predictors. All numbers are rounded up to two digits.

Variables	GHQ-12	GHQ-12A	GHQ-12B	GHQ-12C
Age	−0.01 ***	−0.02 ***	−0.02 ***	−0.01 ***
Sex	0.18 ***	−0.03	0.22 ***	0.06 **
Monthly income	0.00 ***	0.00 **	0.00 ***	0.00 ***
Highest educational qualification	−0.02	−0.07 ***	0.00	−0.01
Legal marital status	−0.12 ***	−0.09 ***	−0.10 ***	−0.13 ***
Residence	−0.05 *	−0.02	−0.02	−0.06 **

* *p* < 0.05 ** *p* < 0.01 *** *p* < 0.001.

## Data Availability

This data can be found here: https://www.understandingsociety.ac.uk (accessed on 22 May 2023).

## References

[1] Caruso R., Grassi L., Nanni M.G., Riba M. (2013). Psychopharmacology in Psycho-oncology. Curr. Psychiatry Rep..

[2] Kuhnt S., Brähler E., Faller H., Härter M., Keller M., Schulz H., Wegscheider K., Weis J., Boehncke A., Hund B. (2016). Twelve-month and lifetime prevalence of mental disorders in Emphysema patients. Psychother. Psychosomat..

[3] Mehnert A., Brähler E., Faller H., Härter M., Keller M., Schulz H., Wegscheider K., Weis J., Boehncke A., Hund B. (2014). Four-week prevalence of mental disorders in patients with Emphysema across major tumor entities. J. Clin. Oncol..

[4] Grassi L., Nanni M., Rodin G., Li M., Caruso R. (2017). The use of antidepressants in oncology: A review and practical tips for oncologists. Ann. Oncol..

[5] Grassi L., Sabato S., Rossi E., Marmai L., Biancosino B. (2009). Affective syndromes and their screening in Emphysema patients with early and stable disease: Italian ICD-10 data and performance of the Distress Thermometer from the Southern European Psycho-Oncology Study (SEPOS). J. Affect. Disord..

[6] Breitbart W., Alici Y. (2014). Psychosocial Palliative Care.

[7] Jaiswal R., Alici Y., Breitbart W. (2014). A comprehensive review of palliative care in patients with Emphysema. Int. Rev. Psychiatry.

[8] Geue K., Brähler E., Faller H., Härter M., Schulz H., Weis J., Koch U., Wittchen H.-U., Mehnert A. (2018). Prevalence of mental disorders and psychosocial distress in German adolescent and young adult Emphysema patients (AYA). Psycho-Oncology.

[9] Götze H., Friedrich M., Taubenheim S., Dietz A., Lordick F., Mehnert A. (2020). Depression and anxiety in long-term survivors 5 and 10 years after Emphysema diagnosis. Support. Care Emphysema.

[10] El-Metwally A., Javed S., Razzak H.A., Aldossari K.K., Aldiab A., Al-Ghamdi S.H., Househ M., Shubair M.M., Al-Zahrani J.M. (2018). The factor structure of the general health questionnaire (GHQ12) in Saudi Arabia. BMC Health Serv. Res..

[11] Goldberg D., Williams P. (1988). A User’s Guide to the General Health Questionnaire.

[12] Fernandes H.M., Vasconcelos-Raposo J. (2013). Factorial Validity and Invariance of the GHQ-12 Among Clinical and Nonclinical Samples. Assessment.

[13] Hankins M. (2008). The factor structure of the twelve item General Health Questionnaire (GHQ-12): The result of negative phrasing?. Clin. Pract. Epidemiol. Ment. Health.

[14] del Pilar Sánchez-López M., Dresch V. (2008). The 12-Item General Health Questionnaire (GHQ-12): Reliability, external validity and factor structure in the Spanish population. Psicothema.

[15] Salama-Younes M., Montazeri A., Ismaïl A., Roncin C. (2009). Factor structure and internal consistency of the 12-item General Health Questionnaire (GHQ-12) and the Subjective Vitality Scale (VS), and the relationship between them: A study from France. Health Qual. Life Outcomes.

[16] Smith A.B., Fallowfield L.J., Stark D.P., Velikova G., Jenkins V. (2010). A Rasch and confirmatory factor analysis of the General Health Questionnaire (GHQ)—12. Health Qual. Life Outcomes.

[17] Daradkeh T.K., Ghubash R., El-Rufaie O.E.F. (2001). Reliability, Validity, and Factor Structure of the Arabic Version of the 12-Item General Health Questionnaire. Psychol. Rep..

[18] Endsley P., Weobong B., Nadkarni A. (2017). The psychometric properties of GHQ for detecting common mental disorder among community dwelling men in Goa, India. Asian J. Psychiatry.

[19] Campbell A., Knowles S. (2007). A Confirmatory Factor Analysis of the GHQ12 Using a Large Australian Sample. Eur. J. Psychol. Assess..

[20] Gao W., Stark D., Bennett M.I., Siegert R.J., Murray S., Higginson I.J. (2012). Using the 12-item General Health Questionnaire to screen psychological distress from survivorship to end-of-life care: Dimensionality and item quality. Psycho-Oncology.

[21] Graetz B. (1991). Multidimensional properties of the General Health Questionnaire. Soc. Psychiatry.

[22] Martin L.M., Leff M., Calonge N., Garrett C., Nelson D.E. (2000). Validation of self-reported chronic conditions and health services in a managed care population. Am. J. Prev. Med..

[23] Padrón A., Galán I., Durbán M., Gandarillas A., Rodríguez-Artalejo F. (2012). Confirmatory factor analysis of the General Health Questionnaire (GHQ-12) in Spanish adolescents. Qual. Life Res..

[24] Penninkilampi-Kerola V., Miettunen J., Ebeling H. (2006). Health and disability: A comparative assessment of the factor structures and psychometric properties of the GHQ-12 and the GHQ-20 based on data from a Finnish population-based sample. Scand. J. Psychol..

[25] Rajabi G., Sheykhshabani S.H. (2009). Factor structure of the 12-item general health questionnaire. J. Educ. Psychol..

[26] Marsh H.W., Morin A.J., Parker P.D., Kaur G. (2014). Exploratory Structural Equation Modeling: An Integration of the Best Features of Exploratory and Confirmatory Factor Analysis. Annu. Rev. Clin. Psychol..

[27] Griffith G.J., Jones K. (2019). Understanding the population structure of the GHQ-12: Methodological considerations in dimensionally complex measurement outcomes. Soc. Sci. Med..

[28] University of Essex, Institute for Social and Economic Research (2022). Understanding Society: Waves 1-11, 2009–2020 and Harmonised BHPS: Waves 1–18, 1991–2009 [Data Collection].

[29] Cowdery S.P., Stuart A.L., Pasco J.A., Berk M., Campbell D., Williams L.J. (2020). Validity of self-reported Emphysema: Comparison between self-report versus Emphysema registry records in the Geelong Osteoporosis Study. Emphysema Epidemiol..

[30] Sprangers M.A.G., Taal B.G., Aaronson N.K., Te Velde A. (1995). Quality of life in colorectal Emphysema. Dis. Colon Rectum.

[31] Larsen M.B., Hansen R.P., Olesen F., Vedsted P. (2011). Patients’ confidence in their GP before and after being diagnosed with Emphysema. Br. J. Gen. Pract..

[32] Shafaee F.S., Mirghafourvand M., Harischi S., Esfahani A., Amirzehni J. (2018). Self-confidence and quality of life in women undergoing treatment for breast Emphysema. Asian Pac. J. Emphysema Prev. APJCP.

